# Epidemiology of complex hand injuries treated in the Plastic Surgery Department of a tertiary referral hospital in Warsaw

**DOI:** 10.1007/s00068-020-01312-5

**Published:** 2020-02-05

**Authors:** Tomasz Dębski, Bartłomiej Henryk Noszczyk

**Affiliations:** Department of Plastic Surgery, Medical Center of Postgraduate Education, Orlowski Memorial Hospital, Czerniakowska 231 Street, 00-416 Warsaw, Poland

**Keywords:** Hand injuries, Reconstructive surgical procedures, Traumatology, Trauma centres, Sick leave

## Abstract

**Background:**

Trauma to the hand is common and potentially serious, impairing daily living and general quality of life. Patients are often unable to work for several months, with hand function improving only gradually. Here, we review the epidemiology of hand injuries treated at a tertiary referral hospital in Warsaw, Poland.

**Material and methods:**

In this single-centre, retrospective study, we reviewed medical records of patients presenting to the A&E Unit of the Plastic Surgery Department, Centre of Postgraduate Medical Education in Warsaw, Poland, between January 2001 and December 2005. We assessed a number of patient and injury characteristics, including severity, scored with the Hand Injury Severity Scoring System (HISS), and time off work.

**Results:**

Of 1091 patients with a hand injury, 84% were male and over half were under the age of 40 years. Hand injury commonly resulted in tendon damage (56.1%), especially to finger flexors (79%), and in skin loss (37.8%). Amputations occurred in 24.1% of cases, while fractures (9.6%) and nerve (6.1%) or joint (5.5%) damage were less common. HISS-graded injury severity was moderate in 28.6% of cases, over half of the patients suffered severe (25.5%) or major (26.5%) injuries, and minor injuries were relatively uncommon (19.4%).

**Conclusions:**

Amongst patients admitted to our Department, the most common injuries were tendon damage, skin loss, and amputations. Over half of the patients presented with severe or major injuries and took six months or longer to return to work, suggesting they were likely to face substantial social and economic consequences of their injury.

**Level of evidence:**

IV: retrospective series.

## Introduction

Traumatic hand injury is common, causing 6.6–28.6% [1–4] of Accident and Emergency (A&E) visits. However, the incidence rates reported in the literature vary widely, ranging from 57.4 per 100,000 [5] to approximately 700 per 100,000 [1]. Amongst the different upper limb injuries—including injuries of the fingers, wrist, lower and upper arm, elbow and shoulder—damage to the fingers is by far the most common; in the United States, an analysis of the National Electronic Injury Surveillance System (NEISS) database showed an incidence of finger injuries that was more than double that of wrist or shoulder injuries, with 444, 181, and 200 people affected per 100,000, respectively [6].

The sequelae of hand trauma can be serious, and patients recover only gradually, with self-assessed hand function, satisfaction in daily occupations, perceived health, and quality of life improving over several months [7]. In a study of 91 patients treated operatively for a hand disorder or injury, the median time to return to work was 10.5 weeks; however, more than half of the patients took longer than 10 weeks to return to work, and 9% took over a year [8]. More intense pain, symptoms of post-traumatic stress disorder, and the injury being work-related were amongst the factors associated with late return to work [8]. Furthermore, patients who sustain more severe injuries tend to take longer to return to work [8–10].

Patients who sustain an acute hand injury experience a temporary or permanent loss of hand function. Thus, these patients generally require some time off work—in the form of sick leave or similar—and may even go on to receive permanent disability benefits. It is, therefore, unsurprising that hand and wrist injuries are associated with substantial costs. They are, in fact, the most expensive injury type, ranking above knee and lower limb fractures, hip fractures, and skull or brain injuries in terms of total costs [3]. The majority of these costs can be attributed to lost productivity rather than healthcare spending [3]. In particular, work-related hand injuries incur higher healthcare and societal costs than injuries occurring during leisure activities [9].

Appropriate and prompt medical intervention restores hand function and reduces the risk of serious sequelae—a study of 419 patients treated for a work-related hand injury at a specialist clinic in India showed that shortening the time between injury and treatment reduces the likelihood of the hand injury resulting in a disability impairing daily living or professional activities [10]. Given the clinical and economic importance of reconstructive treatment in this setting, we aimed to describe the epidemiology of hand injuries encountered in the Warsaw region of Poland, based on the records of patients presenting to the A&E Unit at a specialist Plastic Surgery Department.

## Material and methods

### Study design

This was a single-centre, retrospective study conducted at the Plastic Surgery Department, Centre of Postgraduate Medical Education in Warsaw, Poland. Our hospital is a tertiary referral hospital providing services in the Mazovian district. It encompasses an area of 35,558 km^2^ with a population of 5,356,838. As a referral hospital, we also admit severe hand injury cases from other districts in Poland. Patients admitted to our Department were preselected in Emergency Departments. Admission criteria to our Department were: injury of nerves and vessels, amputations, open tendon injuries isolated or with concomitant bone fractures or nerve injuries, and all injuries with skin defects requiring cover. Isolated fractures, joint lesions, closed tendon injuries, and minor injuries were treated in Orthopaedic Departments or in Emergency Departments. All surgery procedures were performed only by trained plastic surgeons with a minimum of four years of training in plastic surgery.

We reviewed medical records of patients presenting with hand injury at the A&E Unit of our Department between January 2001 and December 2005, and extracted information on patient and injury characteristics. Patient characteristics of interest were age and gender, while injury characteristics included the month in which the injury occurred, the location of the injury within the hand, the type of injury (damage to skin, tendons, joints or nerves, bone fracture, and amputation), and the severity of the injury, assessed using the Hand Injury Severity Scoring System (HISS) [11]. The data was analysed descriptively using Statistica 6.0.

### The HISS scoring system

HISS is a descriptive scoring system specific to hand injuries [11], which is widely used in routine practice. Increasing HISS scores indicate higher severity, with four categories: minor (< 20 points), moderate (21–50 points), severe (51–100 points), and major (≥ 101 points) [11]. The Polish guidelines for the management of severe hand injuries consider it a useful tool for precise determination of injury severity [12]. Importantly, the HISS score correlates with time off work [11] and, used together with the DASH questionnaire, can be used to predict long-term treatment outcome [12].

## Results

Over the five-year study period, 1091 patients presented with a hand injury at the A&E Unit of our Department; 84% of them were male. Over half of the patients were under the age of 40 years, with 20–30 years (30%), 30–40 years (20%), and 40–50 years being the most prevalent age groups. Children and the elderly (aged over 60 years) each represented only 10% of patients or less. Regression analysis showed that the number of cases negatively correlated with the patients’ age (*r* = − 0.5361, *p* < 0.001), which—although not explicitly tested—was especially evident amongst the adult population.

With regard to the time of injury, more patients were admitted during the autumn and winter months, with over 80 admissions per month between September and February and a peak in December (more than 150 cases). Conversely, fewer injuries were seen between March and August, especially in the period April–June (< 60 cases per month).

Surprisingly, work-related injuries occurred most frequently (45.9%). In this group, we also included farm injuries, which constituted the majority of them (75.1%). Home injuries occurred slightly less frequently (38.3%) followed by transport (7.9%), leisure (4.8%), and sport injuries (3.1%). (Table 1).

**Table 1:** Breakdown of accident category

Accident category	*n* (%)
Occupational (including farm accidents)	501 (45.9%) [376 (75,1%)]
Home	418 (38.3%)
Transport	87 (7.9%)
Leisure	54 (4.8%)
Sport	31 (3.1%)
Total	1091 (100%)

Injures of the right hand (50.2%) were only slightly more common than those of the left hand (48.8%), while those affecting both hands were rare, representing only 1% of cases. The majority of injuries affected the digits (86%); hand injuries constituted the remaining 14% of cases. Amongst injuries of the digits, the index finger was most commonly affected (25%), with other fingers representing around 20% of cases each (thumb: 19%, middle finger: 21%, ring finger: 17% and little finger: 18%). The breakdown of injuries by type of structure affected is presented in Table 1.

Tendons were the most frequently damaged structures. (Table 1). Amongst them, flexor tendon damage was by far the most common, representing 79% of cases, with damage to extensor tendons representing the remaining 21%. Finger flexors were most often affected, with tendons of the *flexor digitorum superficialis* and *flexor digitorum profundus* representing 38% of flexor tendon injuries each. Injuries to tendons of other flexors (*flexor carpi radialis*, *flexor carpi ulnaris*, *flexor pollicis longus*, and *flexor pollicis brevis*) jointly represented 24% of cases.

Skin damage was also very common, being present in nearly 40% of cases (Table 1). The vast majority of injuries relating to skin loss affected the digits (94.9%), 44% of which affected the pulp. Only 5.1% of injuries involving skin loss affected the hand.

Approximately a quarter of all hand injuries were amputations (Table 1). Nearly a third of the amputations of the digits involved the index finger (28%), followed by the thumb (23%), ring finger (21%), little finger (17%), and middle finger (11%). Almost half of the amputations were at the level of the distal phalanxes (48%), with 30% at the proximal phalanxes level and 22% at the level of the intermediate phalanxes.

Fractured bones constituted about 10% of damaged hand structures (Table 1). Intra-articular fractures represented 38.7% of fracture cases, followed by diaphyseal (31.6%) and comminuted fractures (29.7%). Regarding the fracture location, 23% affected the thumb, 21% the middle finger, 20% the index finger, 18% the little finger, and 16% the ring finger.

Nerve or joint damage was rare, with each constituting approximately 5% of damaged hand structures (Table 1). Two thirds of joint damage cases involved open wounds (66.6%), while the remaining third were dislocations (33.4%). Nerve damage usually occurred within the digits and the distal portion of the palm, above the level of the carpal tunnel (81%), with only 19% of nerve damage occurring near the wrist. Amongst the digital nerves, those of the middle (27%), index (23%), and little fingers (20%) were most often affected, while innervation of the ring finger (16%) and the thumb (14%) was damaged less frequently.

**Fig. 1:**
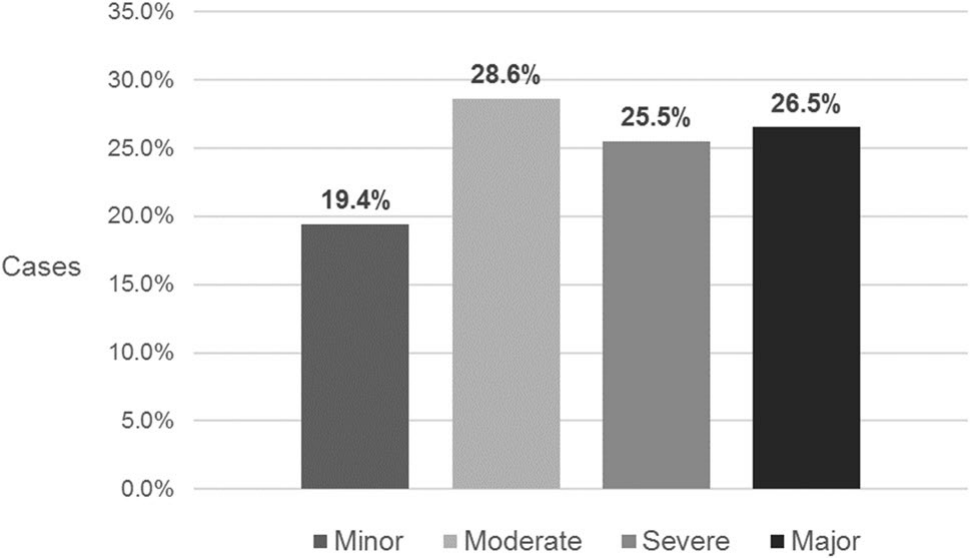
Severity of hand injury cases, according to HISS (*n* = 1091)

**Fig. 2:**
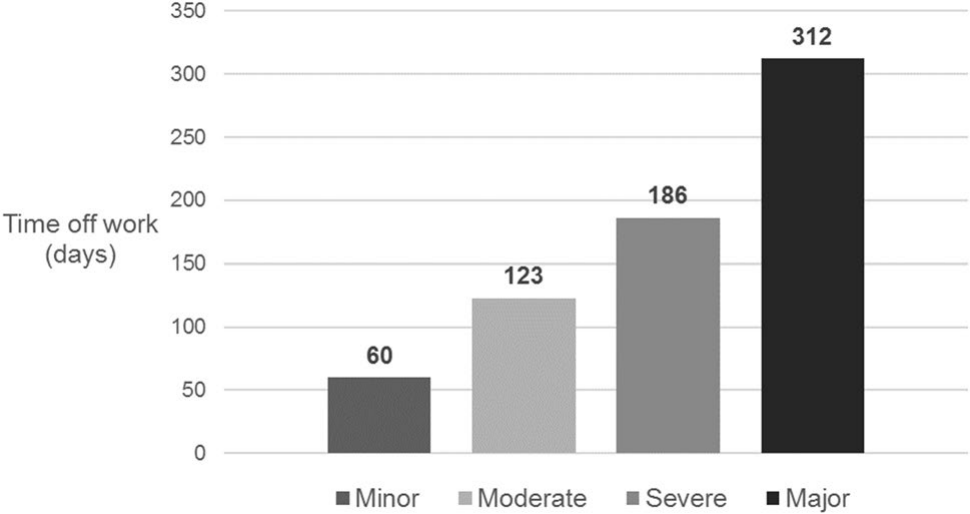
Time off work associated with different levels of injury severity (according to HISS)

Severity of hand injury was classified using HISS [11] (Fig. 1), and the average time off work associated with each severity level was quantified (Fig. 2). Moderate injuries were most common, representing 28.6% of cases; these were associated with an average time off of approximately four months (123 days). While the incidence of severe (25.5%) and major (26.5%) injuries was similar, the average time off work following major injuries was nearly twice as long as that following severe injury. Patients who sustained a severe injury returned to work after approximately 6 months (186 days), while those whose injury was classed as major required more than 10 months (mean 312 days) before becoming fit for work. Minor injuries represented only about a fifth of cases (19.4%), and affected patients were able to return to work after, on average, just two months (60 days).

## Discussion

The majority of patients who presented to the A&E Unit at our Department with a hand injury were young males in active employment, with the frequency of injury decreasing with increasing patient age. Hand injury was more common between September and February, peaking in December. While the reason for this temporal pattern is not entirely clear, it is possible that intensive agricultural work in September and October, agriculture works before winter (e.g. cutting trees for firewood) in autumn and poor weather conditions during the winter months contribute to the increased number of hand injury cases during those periods (Table 2).

**Table 2:** Breakdown of injuries by type

Affected structure	*n* (%)
Tendon damage	1106 (56.1%)
Skin loss	745 (37.8%)
Amputation	475 (24.1%)
Bone fracture	189 (9.6%)
Nerve damage	120 (6.1%)
Joint damage	108 (5.5%)
Total*	2,743 (100%)

* Note that while the number of patients in this study was 1091, the total number of injuries was higher when separated by type of structure affected. This is due to the fact that many patients presented with injuries affecting multiple structures, and these were counted separately in the table

During the study period, we treated a wide variety of hand injuries. With regard to the type of structure affected, these often resulted in finger flexor damage and skin loss. Similar to reports on acute upper extremity injuries from the US [6] and India [10], the fingers were commonly affected in our study. In particular, the tips of the fingers appeared to be prone to amputations, of which nearly half occurred at the distal phalanx level, and skin loss involving the pulp in over 40% of cases.

Notably, over half of the patients we treated suffered an injury classified as severe or major and required, on average, six months or longer before returning to work. Compared with two other epidemiological studies—from Cracow, Poland [5] and Malmo, Sweden [1]—the cases in our study appeared, overall, more severe. Amongst the patients treated for hand injury in Cracow, the most frequently affected group were young, male manual workers, with nearly three-quarters of cases classed as minor or moderate [5]. In Malmo, the majority of affected patients were also young males, but the injuries they sustained were mostly minor (88% of cases) [1]. Only a minority of injuries in the Cracow sample [5] and very few in the Swedish sample [1] were classed as severe (17.1% [5] and 1.8% [1], respectively) or major (7.6% [5] and 0.7% [1], respectively), whereas in our study these injuries were seen in 25.5% and 26.5% of cases, respectively. The large number of patients with severe injuries in our sample results largely from the preselection of patients admitted to our Department and the fact that the Centre of Postgraduate Medical Education in Warsaw is a tertiary referral hospital with experience in treating hand injuries, meaning that numerous patients with severe injuries from all around Poland were referred for treatment at our centre.

Because patients suffering more severe injuries tend to need more time before they can return to work [8, 9, 11], compared with those included in the Cracow and Malmo studies, the patients in our study were likely to experience more prominent social and economic consequences of their injury. In our study, the majority of hand injuries took place during work (occupational accidents). In this group, we also included farm injuries, which constituted the majority of them (75.1%). Most often these injuries were caused by wood-cutting devices such as circular saw, chain saw, or axe as well as agricultural machinery during fieldwork. Injuries usually occurred as a result of the incorrect use of these machines—most often as a result of removing locks and guards protecting against injuries and allowing more efficient work. Smith et al. had similar conclusions, indicating that the most common cause of circular saw injuries was the removal of safety features (riving knife, blade guard, and antikickback) that can hinder the user’s movements [13]*.* Often, wood-cutting machines were also self-made and lacked approvals. Moreover, some of the accidents occurred under the influence of alcohol. In a study by Chow, statistically significant associations were found between the incidence of hand injury and exposure to the following seven factors: using malfunctioning equipment/materials, using a different work method, performing an unusual task, working overtime, feeling ill, being distracted, and rushing [14]. Other data collected in South China indicated that among 2142 patients with hand injuries, 53.85% indicated that “distraction” was the main cause of injury [15].

To prevent work-related injuries, broadly understood education is needed, including the principles of safe use of equipment, improving the protection of power saws [13], prohibition of working on mechanical devices under the influence of alcohol, as well as quality control of the equipment used.

Economic consequences of farm injuries were emphasised in Grandizio’s analysis. He shows that farming remains the most dangerous occupation in the United States, and the mean hospital charges due to upper extremity injuries were $95,147 USD [16].

In addition to the consequences resulting directly from the treatment of injuries, economic and social consequences occur, including income reduction and the need for patient retraining. The health insurance system in Poland awards a pension for complete or partial inability to work, which is a 1/4 of the average salary from previous years, increased by 1.3% for each year of work. Because injuries mainly occur in young people with low work experience, the awarded pension is on average 34% lower than the average monthly salary in Poland (the average pension granted monthly in 2019 was 414 EUR, while the average monthly salary was 1205 EUR—data from the Central Statistical Office, Poland [17]). Reduced income due to inability to work, the need for retraining, and job or residence change (e.g. moving from a village to a city) often cause self-acceptance problems, alcoholism, impoverishment of the family, and other serious social consequences.

To minimise these consequences, emphasis should be put on the workplace, which could reduce the incidence of hand injury by targeting some of its preventable causes.

Furthermore, improving access to specialist hand surgery care could help to promptly and appropriately treat those injuries that cannot be prevented.

Our study offers an insight into the epidemiology of traumatic hand injury in the Warsaw area of Poland and its sequelae. We retrospectively reviewed cases admitted to a tertiary referral hospital and presented information on the characteristics of the patients affected, as well as on the types of hand structures damaged, HISS-graded injury severity, and the time patients required to return to work. One of the main methodological strengths of our study is the relatively large patient sample. However, being a single-centre study from a highly specialised hospital department, our research also has inherent limitations. Notably, the patient sample was probably biased towards more severe cases that would normally be referred to a specialist centre rather than treated at smaller local hospitals. Thus, it is likely that, compared with the total population of hand injury patients in the Warsaw region, patients with minor injuries were under-represented, and those with severe or major injuries were over-represented in this sample. In addition, the relatively dated cohort could be a limitation of our study because the epidemiology of hand injury in the recent period of time could vary. Further investigations are needed to verify changes in hand injury epidemiology after 15–20 years. However, such a relatively dated cohort is very interesting for long-term outcome investigation. Thus, the authors of this paper are currently working on a new study to follow-up the results of hand injury treatment, using DASH/PRWHE and other outcome measurements.

## Conclusion

We described the epidemiology of hand injuries on the basis of 1091 patients presenting to the A&E Unit of our Department. The most common injuries were: tendon damage, commonly affecting finger flexors; skin loss, mainly involving the fingers, especially the pulp; and amputations. Overall, more than half of the patients presented with severe or major injuries, requiring an average of six months or more before becoming fit for work, which suggests that these patients are likely to face substantial social and economic consequences of their injury.
